# A Comprehensive Study on the Amino Acids and Tryptophan-Derived Molecules in Iberian Wine Vinegar

**DOI:** 10.3390/foods13213384

**Published:** 2024-10-24

**Authors:** Catarina Marques, Elisete Correia, Alfredo Aires, Lia-Tânia Dinis, Alice Vilela

**Affiliations:** 1 CITAB, Centre for the Research and Technology of Agro-Environmental and Biological Sciences and Inov4Agro, University of Trás-os-Montes and Alto Douro, 5000-801 Vila Real, Portugal; catarina.ipsmarques@gmail.com (C.M.); alfredoa@utad.pt (A.A.); liatdinis@utad.pt (L.-T.D.); 2 Center for Computational and Stochastic Mathematics (CEMAT), Department of Mathematics, University of Trás-os-Montes and Alto Douro, Apt. 1013, 5001-801 Vila Real, Portugal; ecorreia@utad.pt; 3 CQ-VR, Chemistry Research Center, Department of Agronomy, School of Agrarian and Veterinary Sciences, University of Trás-os-Montes and Alto Douro, 5000-801 Vila Real, Portugal

**Keywords:** HPLC-SPE, amino acids, tryptophan, wine vinegar, health-promoting effect, PCA, HCA

## Abstract

Wine vinegar, valued for its ancient origins and culinary versatility, has garnered scientific interest due to its complex composition and potential health benefits. This study aims to explore the nutritional and bioactive properties of different wine vinegars, focusing on their amino acid content, particularly tryptophan-derived molecules such as serotonin and melatonin. White wine vinegar, red wine vinegar, port wine vinegar, and balsamic vinegar from the Douro and Rioja regions were analyzed using high-performance liquid chromatography and solid-phase extraction (HPLC-SPE). The study examined the amino acid profiles and the presence of serotonin and melatonin across the samples. The analysis revealed the presence of significant bioactive amino acids, including arginine (found in sample 059 at 61.21 mmol/L), alanine (in a concentration of 30.33 mmol/L in sample 209), and threonine (sample 336 presented the highest concentration—71.47 mmol/L), which have been linked to cardiovascular health, immune system support, and mucosal regulation. The amino acid content varied among the vinegar types, with slower acetification and prolonged aging reducing their concentrations. Tryptophan was mainly found in sample 059 (30.54 mmol/L). These findings, with their potential to influence the scientific community’s understanding of the health-promoting properties of wine vinegar, particularly its amino acid content and the potential influence of production processes on bioactive molecules, are of great interest.

## 1. Introduction

Vinegar is a versatile elixir revered for its gustatory contributions and potential health benefits. Wine vinegar emerges as a prominent contender among its myriad forms, tracing its origins back to ancient civilizations. Originating predominantly from the Iberian Peninsula, wine vinegar has transcended its culinary role to become a subject of scientific inquiry due to its complex composition and purported health-promoting properties.

Functional compounds in vinegar enhance flavor and are essential in preventing and treating human diseases through their antibacterial and anti-inflammatory properties [1]. Several authors suggest that bioactive compounds in food may reduce incidences of degenerative illnesses by supplying an antioxidant effect [2,3]. Recent literature reinforces the role of fermented foods and bioactive food compounds in maintaining the integrity of the intestinal barrier, shaping microbiota, alleviating inflammation, and restoring homeostasis [4] and their association with the prevention of diseases, for example, lowering the risk of type 2 diabetes mellitus [5].

The amino acid (a.a.) compounds in vinegar reinforce the effectiveness of vinegar’s health benefits. They are bioactive compounds that improve immunity [6], promote brain development, and protect from neurodegenerative diseases. They are also essential nutrients for cellular regulation [7]. They also directly impact the central nervous system and muscle energy supply [8].

These compounds have been successfully determined by gas chromatography coupled with mass spectrometry (GC-MS) and by high-performance liquid chromatography (HPLC) coupled with electrochemical, UV, and fluorescence detectors [9,10,11]. According to Muñiz-Calvo and coworkers [12], there are other techniques to detect these molecules, such as HPLC with capillary electrochromatography, thin-layer chromatography coupled with densitometric detection, ultrahigh performance liquid chromatography coupled with high-resolution tandem mass spectrometry, radioimmunoassay (RIA) and enzyme-linked immunosorbent assay (ELISA).

Specific literature on a.a. composition in wine vinegar is scarce. Mutaguchi and coworkers [13] analyzed the distribution of d-amino acids in vinegar (grain, fruit, and vegetable vinegar); however, the results are not representative as they only studied one white wine vinegar and one balsamic vinegar. Chinnici and coworkers [14] also quantified twenty-three a.a. in 37 premium-quality European vinegars but only approached the balsamic vinegar of Modena.

Tryptophan-derived molecules, like melatonin and serotonin, are present in wine at deficient concentrations, varying from picograms to ng/mL; the most suitable detection method is HPLC-SPE [15]. Concentrations of tryptophan-derived molecules in wine and beer are deficient but sufficient for dietary intake to measure their effects through various methods, with yeasts playing a clear role in their formation [7,16]. However, there is a lack of literature regarding identifying and quantifying these specific molecules in wine vinegar.

Moreover, a.a. vinegar composition also depends on the vinegar production process. The submerged method employs a Frings acetifier. This method accelerates the acetification process by optimizing oxygenation and temperature control, ensuring efficient ethanol conversion into acetic acid [17,18]. The submerged method allows for rapid production while maintaining consistent quality. Callejón and coworkers [19] discussed submerged acetification and compared it to surface methods, highlighting that the consumption of amino acids is much lower in submerged than in surface acetifications. Studies have shown that submerged acetification, particularly with modern equipment like the Frings acetifier, enhances the efficiency of acetic acid bacteria by providing optimal aeration and temperature management, which is crucial for maintaining the quality of the final product [17,18]. Furthermore, this method ensures consistent and reproducible results, making it ideal for large-scale vinegar production.

In contrast, balsamic vinegar (BV) is produced using a modified Orleans method explicitly adapted for balsamic vinegar production. This traditional method involves slow acetification by acetic acid bacteria in different wooden barrels. The prolonged fermentation and aging process in wooden barrels contributes to balsamic vinegar’s complex aromatic and flavor profiles [20,21].

Port Wine Vinegar (PWV) follows a unique process developed in the wine cellars where it is produced. Initially, port wine is diluted with potable water to reach an alcohol content of approximately 13–16% (*v/v*). Subsequently, a Frings acetifier is used to accelerate the acetification process. Finally, the acetified wine is aged in wooden casks, allowing the typical woody characteristics of port wine to be transferred into the vinegar.

This research aims to assess white, red, balsamic, and port wine vinegar from different regions and manufacturing processes by measuring the amino acid content by HPLC-SPE, focusing on bioactive compounds with health-promoting properties.

## 2. Materials and Methods

### 2.1. Samples

Two different regions were selected for the study: La Rioja in Spain and the Douro Demarcated Region (DDR) in Portugal. The study used twenty-two vinegar samples (Table 1): fifteen from the DDR and seven from Rioja. Eleven vinegar samples were processed in the DDR subregion Cima Corgo and four in the Douro Superior subregion; all Spanish vinegar samples were from Rioja Alta.

The vinegar from the Douro Region (Cima Corgo and Douro Superior), including port wine vinegar, was made from wines vinified with the Douro region grape varieties Bastardo, Mourisco tinto, Tinta Amarela, Tinta Barroca, Tinta Cão, Tinta Roriz (the same as Spain’s Tempranillo), Touriga Francesa and Touriga Nacional (red wine vinegar), and the white grapes Donzelinho branco, Gouveio, Malvasia Fina, Rabigato, and Viosinho (white wine vinegar).

The predominant soil in the Cima Corgo region is schist, a type of metamorphic rock. The soil is generally shallow, poor in organic matter, and composed of fragmented schist layers. The Cima Corgo enjoys a Mediterranean climate characterized by hot, dry summers and mild, wet winters. The steep, terraced vineyards of Cima Corgo create various microclimates depending on factors like altitude, sun exposure, and proximity to the Douro River. Altitude is crucial in moderating temperatures, as higher-altitude vineyards tend to be cooler, producing fresher, more elegant wines.

Like the other Douro subregions, the Douro Superior has predominantly schist soils. Granite soils are present in certain parts of the Douro Superior, especially at higher altitudes. Granite tends to be more acidic and less fertile than schist, impacting vine growth and wine style in those areas. Unlike the more temperate Baixo Corgo and Cima Corgo regions, the Douro Superior has a more extreme continental climate. This means that temperatures can often soar above 40 °C (104 °F) during the summer months, making it one of the hottest wine-growing regions in Europe. The Douro Superior is much drier than the Cima Corgo. Annual rainfall can be as low as 400–500 mm, making drought stress a significant challenge for viticulture. However, this dry environment also reduces the risk of disease, which can plague vines in more humid regions. The terrain is generally flatter and more expansive than the Cima Corgo. Higher-altitude vineyards in the Douro Superior can experience slightly cooler temperatures, which can be beneficial for preserving acidity and freshness in the grapes.

Rioja Alta red wine and balsamic vinegar were made from Tempranillo, Graciano, and Garnacha grapes. At the same time, white wine vinegar (sample 876) was made from Viura (also known as Macabeo), Malvasía, and Garnacha Blanca grape varieties.

The terroir of Rioja Alta is distinct from the neighboring Rioja Alavesa and Rioja Oriental (formerly Rioja Baja) primarily due to its altitude, soil composition, and climate. One of the critical soil types in Rioja Alta is calcareous clay, which is ideal for growing Tempranillo. This soil has a good water retention and drainage balance, which helps sustain the vines during dry spells without causing waterlogging. Alluvial soils (composed of sand, silt, and gravel) are found in the lower areas near rivers.

Rioja Alta benefits from a continental climate with a significant Atlantic influence, which moderates the temperatures and brings some humidity and rainfall. This is in contrast to the warmer, drier conditions found in the Rioja Oriental.

Rioja Alta is located at a higher altitude (often around 500–700 m above sea level) than Rioja Oriental. The elevation helps to moderate the temperatures, providing a longer growing season crucial for developing complexity and structure in the wines.

The production methods for the different vinegars analyzed in this study vary according to their origin and techniques (Table 1). For White Wine Vinegar (WWV) and Red Wine Vinegar (RWV), the submerged method employed the Frings acetifier. Balsamic vinegar was made using the traditional Orleans Method, adapted for balsamic vinegar production. Port wine vinegar is obtained by applying submerged acetification (Frings acetifier) followed by aging in wood, usually French or Portuguese oak wood barrels.

### 2.2. Chemicals and Reagents

All reagents were purchased from Sigma-Aldrich (Taufkirchen, Germany). Methanol (cas number 67-56-1), sodium acetate (cas number: 127-09-3), TFA (trifluoroacetic acid, cas number: 76-05-1), OPA (o-phthalaldehyde, cas number: 643-79-8), mercaptoethanol (cas number: 60-24-2), potassium borate (1M, pH 9.5, cas number: 14680-77-4), aspartic acid (cas number: 56-84-8); glutamic acid (cas number: 56-86-0); asparagine (cas number: 70-47-3); serine (cas number: 56-45-1); histidine (cas number: 71-00-1); glutamine (cas number: 56-85-9); glycine (cas number: 56-40-6); threonine (cas number: 72-19-5); arginine (cas number: 74-79-3); alanine (cas number: 56-41-7); tyrosine (cas number: 60-18-4); valine (cas number: 72-18-4); methionine (cas number: 63-68-3); tryptophan (cas number: 73-22-3); phenylalanine (cas number: 63-91-2); isoleucine (cas number: 73-32-5); leucine (cas number: 61-90-5); melatonin (N-acetyl-5-methoxytryptamine, cas number: 73-31-4); serotonin (5-hydroxytryptamine, cas number: 50-67-9).

### 2.3. Total Amino Acids Content Extraction

The wine vinegar samples were prepared using an SPE (solid-phase extraction) procedure based on the methodology proposed by Albu and coworkers [15]. To activate the SPE cartridges (Bond Elut C18, 500 mg, and 3 mL, Agilent), 2 mL of methanol followed by 2 mL of Milli-Q water were used. Then, the sample (1 mL) was loaded and washed with 1 mL of methanol. Later, samples were thoroughly dried and redissolved in 0.2 mL of methanol. At last, samples were centrifuged for 5 min at 12,500 rpm, and the supernatant was transferred into filter units (0.2 μm PTFE membrane filter), filtered, and injected into the HPLC system. The results were expressed as mmol/L for the amino acids.

### 2.4. Amino Acids and Tryptophan-Derived Molecules Analysis by HPLC

The HPLC-DAD-UV-Vis analysis of free amino acids was performed on a Gilson system equipped with a C18 column (stationary phase) fitted with a C18 column (150 × 4.6 mm, 5 µm), a diode array detector and a binary gradient composed of a solvent A (sodium acetate 0.1M pH 6.95 plus methanol and TFA in a 92.5:5:2.5 ratio), a solvent B (methanol and TFA in a 97.5:2.5 ratio) with an elution gradient of 0 min—0% solvent B; 6 min—10% solvent B; 8 min—15% solvent B; 12 min—25% solvent B; 15 min—40% solvent B; 18 min—40% solvent B; 20 min—60% solvent B; 24 min—100% solvent B; 24.5 min—100% solvent B; 26 min—0% solvent B and 37 min—0% solvent B.

The flow rate used was 1.2 mL min^−^¹, with the column at room temperature (±22 °C) and an injection volume of 10 µL. The chromatograms were recorded in the 220 to 600 nm range, and the compounds were detected at a wavelength of 340 nm. Prior the HPLC injection, the samples were derivatized in a mixture of 6.3 mg OPA (o-phthalaldehyde, cas number: 643-79-8, Sigma-Aldrich, Taufkirchen, Germany) with 250 µL methanol, 6.5 µL mercaptoethanol (cas number: 60-24-2 Sigma-Aldrich, Taufkirchen, Germany) and 21.5 mL potassium borate 1M, pH 9.5 (cas number: 14680-77-4 Sigma-Aldrich, Taufkirchen, Germany). For derivatization, 20 µL of each sample or standard was added to 50 µL of the derivatization solution, left to react for 2 min, and then injected into HPLC. Identification was performed based on a pre-run of free amino acid standards, their retention times, and spectra. Quantification was done using external calibration curves, and results were expressed in millimoles/L.

### 2.5. Statistical Analysis

Descriptive measures, such as mean (M) and standard deviation (SD), were calculated. Skewness and kurtosis coefficients were computed for univariate normality analysis purposes, and all values were within ±1. Multivariate analysis of variance (MANOVA) followed by, whenever possible, one-way analysis of variance (ANOVA) and Tukey’s post hoc test were used to investigate differences between vinegar samples on the analyzed amino acids. A principal component analysis (PCA) was carried out to identify and understand the structure of the most explanatory variables (amino acid compounds) in each component. An eigenvalue greater than one was used as the criterion for retaining components. A Hierarchical Cluster Analysis (HCA) was performed to examine the similarities and differences between vinegar and the amino acids studied. Ward’s method with squared Euclidean distance was applied to the average sample profiles, and the results were visually represented in a dendrogram. Notice that in some vinegar samples, no amino acids were detected (587, 398, 927, 809, 101, 215, 198, 703, and 613).

All statistical analyses were conducted using the R package (R-4.4.1 for Windows) and Excel (Microsoft 365). A significance level of 0.05 was considered.

## 3. Results and Discussion

### 3.1. Total Amino Acid Content

The results for the amino acid composition of wine vinegar samples are shown in Table 2.

According to Chinnici and coworkers [14], many factors can influence the presence of amino acids in vinegar. These factors can be influenced by the selected production process and its biological evolution. Despite the impact of these factors on the total amino acid content of vinegar, the literature attempts to distinguish different vinegar categories through their amino acid content [14,22].

Table 2 presents the total amino acid content in different wine vinegar varieties: white wine vinegar, red wine vinegar, balsamic vinegar, and port wine vinegar. Testing was conducted based on statistical differences in the concentration of amino acids among the different samples, ensuring that the observed variations were statistically significant.

The analysis of Table 2 revealed significant variations, with sample 059 (WWV) exhibiting notably higher concentrations than all remaining samples. Amino acid concentration in beverages can be increased through several methods. The direct addition of isolated amino acids allows precise control over their types and amounts, enhancing the beverage’s nutritional profile. Protein hydrolysates, derived from enzymatically broken-down proteins, are also used to boost amino acid content due to their rapid absorption and utilization, making them ideal for functional drinks [23]. Enrichment with naturally rich ingredients and fortification with amino acid supplements are also effective strategies [24,25]. Adjusting the pH of the beverage can improve the stability and solubility of amino acids, ensuring their bioavailability [26]. Stabilization and encapsulation technologies protect amino acids during processing and storage, maintaining their concentration in the final product [27]. Fermentation using specific bacteria can naturally enhance the amino acid content of beverages by breaking down proteins into amino acids during fermentation [28].

The stark differences in amino acid concentrations between sample 059 and the other samples can be mainly attributed to the source and production methodology of the samples. Sample 059 was acquired from a supermarket, while the remaining samples were obtained directly from producers. This difference in sourcing likely accounts for the observed variations due to several factors. Firstly, the supermarket sample (059) underwent different handling and processing protocols to the producer samples. Industrial-scale production and packaging for mass-marketed products often include additional steps or additives that help preserve amino acids. This is supported by Budak and coworkers [29], who describe various industrial processes and additives used in vinegar production that help maintain its functional properties, including amino acid preservation. Secondly, sample 059 was produced using submerged acetification, a method commonly employed in mass production to expedite the fermentation process [19]. As detailed by Budak and coworkers [29], this method involves improved aeration and stirring, which can result in higher preservation of bioactive components, including amino acids, compared to traditional surface methods used by local producers. Lastly, supermarket products are stored under controlled conditions designed to maximize shelf life and maintain quality. Industrial-scale production and packaging for mass-marketed products often include additional steps or additives that help preserve amino acids [19]. These conditions likely contribute to the higher concentrations of amino acids observed in sample 059. By comparing the different categories of wine vinegar, it is observed that BV is the poorest category in terms of the total amount of amino acids, followed by PWV.

The amino acids analyzed in this study were chosen for their known bioactive properties, such as supporting cardiovascular health, immunity, and mucosal regulation [6,7,8]. Cysteine was not included in the study due to limitations related to the equipment and methodology. The HPLC system and the derivatization process used in the lab tend to degrade or fractionate cysteine significantly, making its accurate detection and quantification unreliable. As for lysine, although it is essential for human health, lysine is typically present in lower concentrations in plant-based foods than in animal-based sources. Many staple crops, like cereals, are naturally low in lysine content, which is a significant limitation for plant-based diets. This is why lysine often becomes the limiting amino acid when people rely heavily on non-animal protein sources. For this reason, it is necessary to supplement plant-based diets with lysine-rich ingredients to meet nutritional needs [30]. Given these factors, including lysine in the analysis with the current setup was not feasible.

Arginine is an amino acid that has proved effective as a health-promoting compound. It enhances nitric oxide, improving inflammatory and oxidative processes in the vascular endothelium, and may be crucial in the development of atherosclerosis. It can be produced endogenously by our body or naturally found in some plants and food [31]. Additionally, arginine improves circulation and immunity, effectively lowers blood pressure, reduces inflammation, prevents infections [32], and treats erectile dysfunction [33]. Its mean intake varies according to the population’s demographic and cardiovascular risk factors [34]. According to Cynober and coworkers [35], young adults should not exceed a mean intake of 30 g/day. In this study, the mean arginine concentration was 5.56 mmol/L, and a higher arginine concentration was found in sample 059 of WWV (61.21 mmol/L). In Chinnici and coworkers [14], the average concentration of arginine in Jerez vinegar and balsamic vinegar of Modena ranged from 11.72 to 89.15 mg/kg, exhibiting a variation as in our study.

Another popular amino acid for its health properties, especially among athletes, is alanine. It can be found in meat and poultry and is responsible for increasing carnosine levels in human skeletal muscle, augmenting the energy for muscles and the central nervous system. This non-proteogenic amino acid strengthens the immune system and helps the body use sugars [6,8]. The mean concentration in wine vinegar samples was 5.44 mmol/L; a higher concentration was noticed in the sample of RWV 209 (30.33 mmol/L). For this amino acid, the average concentration in the literature ranged from 22.98 to 212.82 mg/kg [14].

Threonine is also an essential amino acid with multiple health benefits. It assists in maintaining the proper protein balance in the body and is necessary to regulate mucosal integrity [36] and collagen formation [37]. Regarding wine vinegar samples in this study, threonine was found in a mean concentration of 3.88 mmol/L, and the sample of WWV 059 presented the highest concentration (71.47 mmol/L). The average concentration for this amino acid in the literature ranged from 12.48 to 128.56 mg/kg [14].

Even in categories like BV, where amino acid content is near zero, the only amino acids detected in deficient concentrations in sample 326 were arginine, alanine, and threonine (1.47, 0.40, and 0.79 mmol/L, respectively).

**Figure 1:**
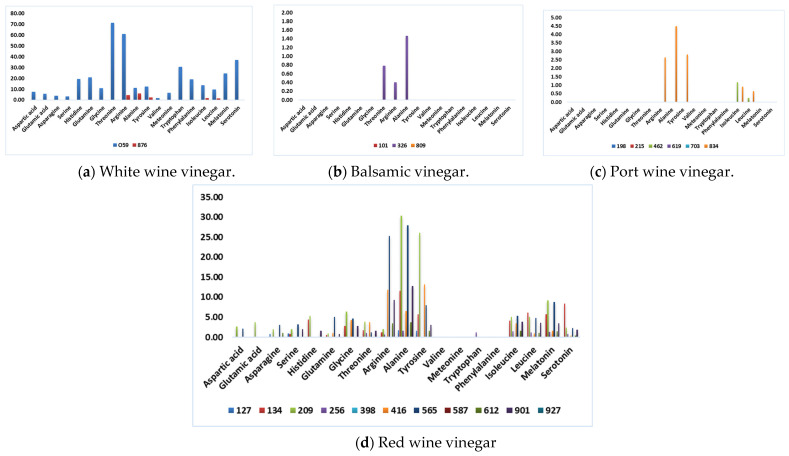
Amino acid composition (mmol/L), identified through HPLC analysis.

Figure 1 presents the data by considering each type of wine vinegar. It illustrates each type’s differences and specific characteristics, allowing for a clear and intuitive visual analysis of the information.

The analysis of Figure 1 shows that BV and PWV are the wine vinegar categories with the lowest amino acid content. This conclusion validates Chinnici and coworkers’ [14] theory that lower amino acid content was found in slower acetification processes and extended wood aging of vinegar. They attribute this phenomenon to Maillard reactions that could influence the decrease of amino acids.

In contrast, sample 059 has the highest total content of amino acids. This WWV is mass-marketed to a large retail group and produced by submerged acetification. Although this vinegar has numerous amino acids with direct human health benefits, it is impossible to affirm that a specific mean daily intake of this vinegar will bring those benefits, as several authors still need to propose a daily intake of some of these amino acids [38,39]. Nevertheless, the therapeutic effect of a daily drink containing 15 mL vinegar (750 mg of acetic acid) was reported, giving the responsibility to the presence of acetic acid and other components in vinegar [3].

The improvement of the glycemic response to carbohydrate-rich meals seems to be linked with daily vinegar intake in amounts of ~10–30 mL (~2–6 tablespoons) [40]. Taking that amount and the total amino acid content of sample 059 into consideration, Table 2 presents the daily intake of each amino acid. In other words, if consumers use around 10mL of vinegar (two tablespoons) daily, their intake will be comparable to the values in Table 3.

### 3.2. Statistical Grouping Study

**Figure 2:**
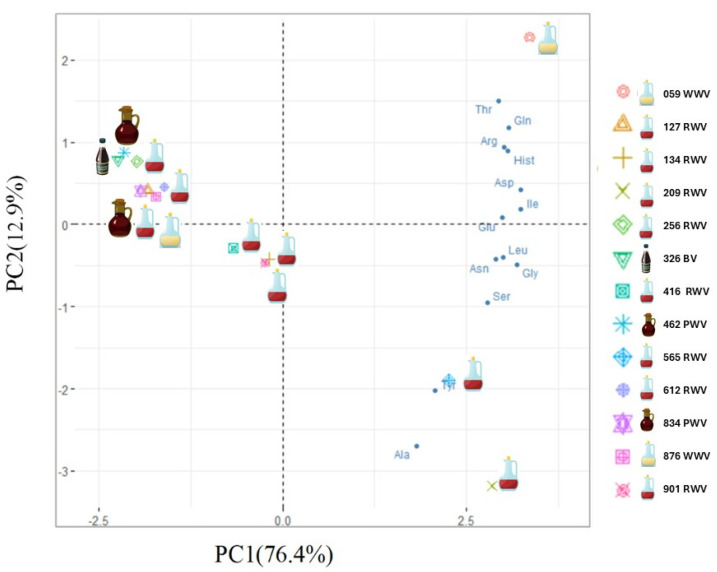
Biplot (PC1 vs. PC2) of PCA model built with wine vinegar’s total amino acid content data.

To identify the structure of relationships among the observed data, a principal component analysis (PCA) was performed. According to the eigenvalue greater than one rule, the correlational structure of the data is explained by two components. The correlations between variables highlight the first component (PC1), mainly explained by aspartic acid, glutamic acid, isoleucine, leucine, glycine, and asparagine, and the second component (PC2), more explained by threonine and alanine. The first principal component explains 76.42% of the total variance, and the second explains 12.90% (Figure 2).

Additionally, a biplot was created to see how variables and wine vinegars relate (Figure 2). As can be noticed, no clear pattern was observed in the samples based on the variety. However, this graphical representation suggests three groups of wine vinegars. Sample 059 (WWV), in the upper right corner, clearly distinguishes itself from the remaining samples, mainly explained by threonine, glutamine, arginine, and histidine, and samples 209 and 565 (both RWV from Rioja Alta), in the lower right quadrant, are more described by alanine and tyrosine. The wine vinegar samples 127, 134, 256, 416, 612, 901 (all RWV), 876 (WWV), 326 (BV), 462, and 834 (both PWV) have the lowest amino acid content and form a group in the left of the PCA graphical representation.

**Figure 3:**
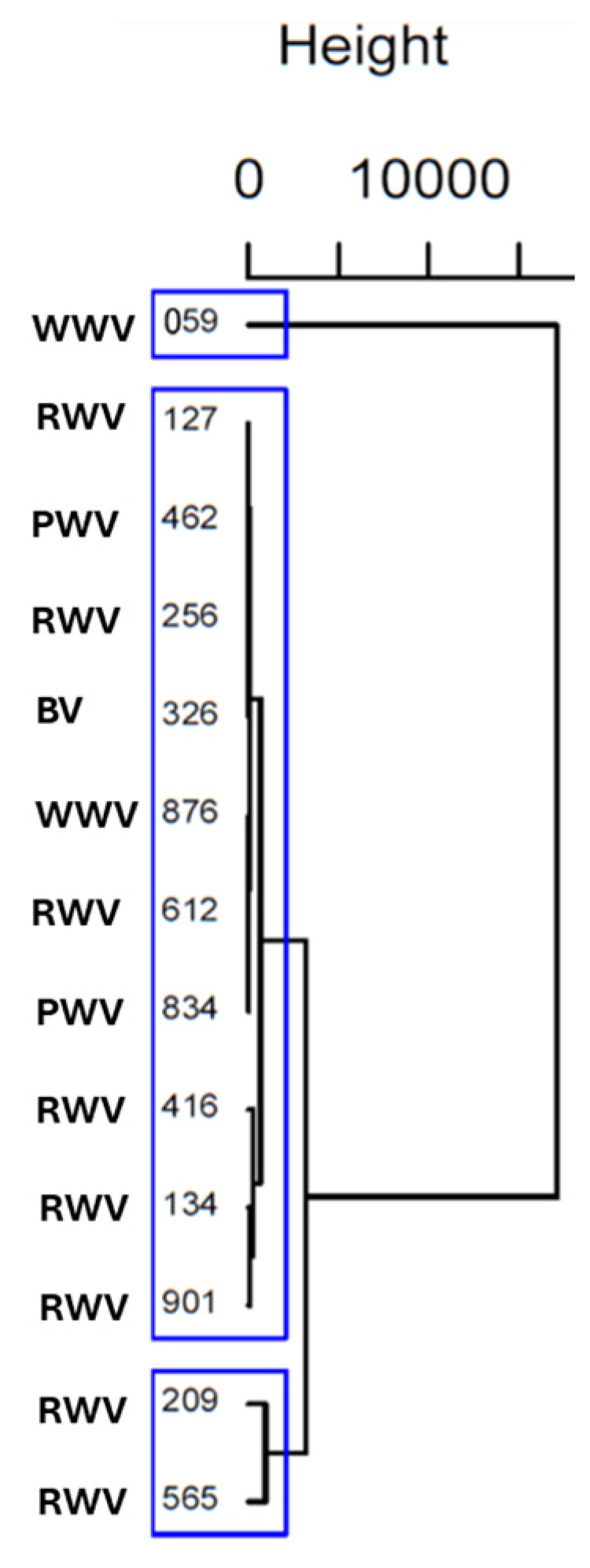
Dendrogram from HCA using Ward’s methodology and Euclidian Squared distance, considering amino acid content of wine vinegar samples. The blue boxes revealed three distinct clusters.

An HCA was performed to thoroughly examine these differences and find homogeneous subgroups among vinegar samples. Ward’s method with squared Euclidean distance was applied. The results were visually represented in a dendrogram (Figure 3).

The cluster analysis revealed that vinegar samples 127, 462, 256, 326, 876, 612, 834, 134, and 901, independently of their origin (terroir) and type of vinegar (white, red, port or balsamic), share similar characteristics; vinegar samples 209 and 565 (RWV from Rioja Alta) also have similar characteristics. On the other hand, the vinegar sample of 059 (WWV) does not share characteristics identical to those of the others in terms of amino acids. Therefore, the HCA results revealed three distinct clusters of vinegar samples that align well with the patterns identified in the PCA, providing consistent and reinforcing insights into the underlying similarities and differences among the samples.

The grouping of wine vinegar samples in PCA and HCA does not reflect underlying similarities in their production methods or origin (terroir); the large group presents vinegar samples from both regions (Douro and Rioja Alta), diverse grape varieties (white and red grapes), and distinct fermentation conditions (in the same large group we have red, white, port and balsamic vinegar samples).

However, our data and the cited literature suggest that production factors, like the acetification process, may influence the amino acid profile of the vinegar samples. For example, WWV (mainly sample 059) produced through submerged acetification tends to have higher amino acid concentrations than BV or PWV. In this rapid acetification method, producers add nutrients, like nitrogen, to facilitate the acetification. It also allows for better oxygenation and temperature control, preserving amino acids that may otherwise be consumed by the acetic acid bacteria (AAB) during slower, surface-level fermentations. In contrast, vinegar aged in wooden barrels, such as BV and PWV, show lower amino acid content. This is likely due to the consumption of nitrogen in the initial wine by the AAB concomitant with Maillard reactions during prolonged aging, which decreases amino acid levels [42,43].

Despite our data, terroir, including soil nitrogen levels, may also play a significant role. Vinegar from grapes grown in soils with higher nitrogen content could yield vinegar with a richer amino acid profile, as nitrogen is crucial for amino acid synthesis during grape development. The differences in soil quality between regions, like La Rioja and Douro, likely contribute to some of the variation in amino acids. Clay soils, also found in Rioja Alta, are rich in nitrogen [44], which benefits wines in several ways. Grapevines use nitrogen to build essential compounds, including proteins, enzymes, nucleoid acids, pigments, and amino acids. So, this can be one explanation for the fact that 209 and 565 red wine vinegar samples from Rioja Alta contain a higher amount and significant diversity in amino acids, as observed in Table 2. Grapes from nitrogen-poor soils might lead to vinegar with fewer amino acids, while those from nitrogen-rich soils could produce higher amino acid content [43].

### 3.3. Tryptophan-Derived Molecules

Tryptophan-derived molecules like melatonin and serotonin have become relevant in food and beverage. Therefore, these molecules have been highlighted in this study. Besides health-promoting properties, these compounds are essential nutrients regulating cellular metabolism. Melatonin (MEL) is a neurohormone (n-acetyl-5-methoxytyramine) from the pineal gland produced as a secondary metabolite in the plant kingdom. It is produced by *O*-serotonin methylation followed by n-methoxytryptamine acetylation in yeast and can also be synthesized from tryptophan, 5-hydroxytryptophan, serotonin, and ultimately n-acetylserotonin. This molecule is a powerful antioxidant and is very efficient in re-establishing the circadian rhythm, helping in sleeping disorders. It protects against neurodegenerative diseases like Parkinson’s, Alzheimer’s, Huntington’s, amyotrophic lateral sclerosis, and fibrinogenesis [7]. Serotonin (SER) is formed during malolactic and alcoholic fermentation by the action of yeast and lactic acid bacteria. It derives from the decarboxylation of L-tryptophan [15]. Serotonin is a neurotransmitter that plays a crucial role in mood regulation, sleep, and other physiological functions, as it is a vital mediator in fibrosis and vasculopathy, helping in diseases like systemic sclerosis [45].

**Figure 4:**
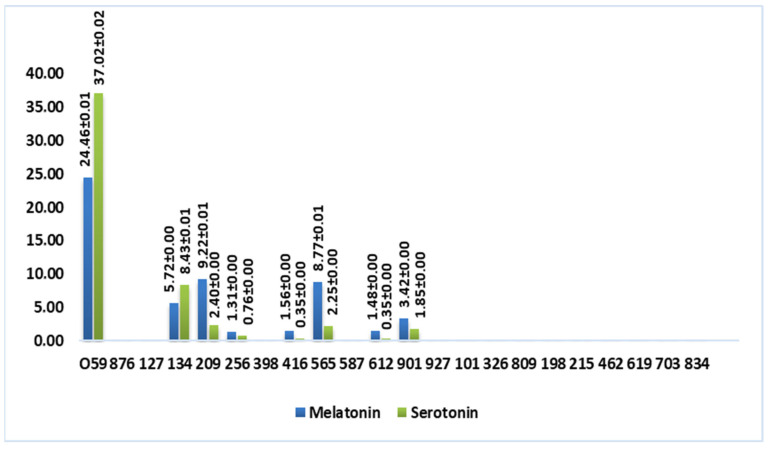
Total content of tryptophan-derived molecules in wine vinegar (mmol/L).

Figure 4 shows a graphic representation that better visualizes the content of tryptophan-derived molecules in wine vinegar samples (mmol/L).

According to Figure 4, these molecules were not detected in balsamic and port wine vinegar, which suggests that the aging process impacts the compounds’ stability. Albu and coworkers [15] observed the same phenomenon when analyzing different Romanian wines.

Interestingly, white wine vinegar sample 059 exhibited more than double the melatonin content (24.46 mmol/L) of any other sample and nearly four times the serotonin amount (37.02 mmol/L). Additionally, some red wine vinegar samples (134, from Douro Cima Corgo; 209 and 565, from Rioja Alta) showed an average melatonin concentration of 7.90 mmol/L and serotonin concentration of 4.36 mmol/L. These results indicate that the type of vinegar and specific production methods, in this case, submerged fermentation, may play a crucial role in determining the content of these compounds.

## 4. Conclusions

This research explored wine vinegar samples’ intricate composition and potential health-promoting properties. A meticulous analysis of total amino acid content, particularly the presence of tryptophan-derived molecules, uncovered significant insights into the nutritional profile of various wine vinegar categories.

Our findings underscored the significant role of amino acids such as arginine, alanine, and threonine in wine vinegar, highlighting their potential health benefits ranging from cardiovascular support to immune system enhancement. The differential presence of these amino acids across vinegar categories shed light on the influence of production processes, with slower acetification and extended wood aging potentially diminishing amino acid content. The analysis revealed that sample 059, sourced from a supermarket, had significantly higher amino acid concentrations than other samples, likely due to different handling, processing, and storage conditions.

Moreover, the absence of tryptophan-derived molecules like melatonin and serotonin in specific vinegar categories, like balsamic and port wine vinegar, hinted at the impact of aging processes on compound stability. The study highlighted the role of production processes and aging in influencing amino acid levels and opens avenues for further research into the effects of aging techniques on the retention of bioactive compounds in vinegar.

## Figures and Tables

**Table 1:** Type of vinegar, sample code, and source (sub-region) of vinegar samples used in the work and production process. Each sample had two replications.

Type of Vinegar	Sample Code	Source (Sub-Region)	Production Process
WWV ^1^	876	Producer (Rioja Alta)	Frings acetifier (submerged method)
	059	Supermarket	
RWV ^2^	134	Producer (Cima Corgo)	Frings acetifier (submerged method)
	256	Producer (Cima Corgo)	
	587	Producer (Cima Corgo)	
	398	Producer (Cima Corgo)	
	927	Producer (Cima Corgo)	
	416	Producer (Douro Superior)	
	127	Producer (Douro Superior)	
	901	Producer (Douro Superior)	
	612	Producer (Rioja Alta)	
	209	Producer (Rioja Alta)	
	565	Producer (Rioja Alta)	
BV ^3^	809	Producer (Rioja Alta)	Modified Orleans method
	101	Producer (Rioja Alta)	
	326	Producer (Rioja Alta)	
PWV ^4^	834	Producer (Douro Superior)	Frings acetifier (submerged method) and aging in wood
	215	Producer (Cima Corgo)	
	462	Producer (Cima Corgo)	
	198	Producer (Cima Corgo)	
	703	Producer (Cima Corgo)	
	619	Producer (Cima Corgo)	

^1^ White Wine Vinegar, ^2^ Red Wine Vinegar, ^3^ Balsamic Vinegar, and ^4^ Port Wine Vinegar.

**Table 2:** Amino acid compounds (mmol/L) in wine vinegar are affected by varieties (white wine vinegar—WWV, red wine vinegar—RWV, balsamic vinegar—BV, and port wine vinegar—PWV). Mean and standard deviation are represented in the same column, and the results with different letters mean they are statistically different from each other (*p* < 0.05).

	Sample	Asp	Glu	Asn	Ser	Hist	Gln	Gly	Thr	Arg	Ala	Tyr	Val	Met	Tryp	Phe	Ile	Leu
**WWV**	**876**	n.d.	n.d.	n.d.	n.d.	n.d.	n.d.	n.d.	n.d.	4.48 ±0.04 ^f^	6.18 ±0.08 ^d^	2.29 ±0.02 ^b^	n.d.	n.d.	n.d.	n.d.	1.78 ±0.01 ^d^	1.46 ±0.01 ^e^
	**059**	7.66 ±0.09 ^c^	5.71 ±0.07	3.86 ±0.05 ^e^	3.31 ±0.05 ^d^	19.33 ±0.12 ^d^	21.06 ±0.17 ^e^	11.01 ±0.09 ^e^	71.47 ±0.34 ^f^	61.21 ±0.30 ^j^	11.33 ±0.12 ^f^	12.57 ±0.12 ^f^	1.74 ±0.04	6.64 ±0.07	30.54 ±0.19 ^b^	19.11 ±0.17	13.61 ±0.15 ^j^	9.64 ±0.11 ^j^
**RWV**	**134**	n.d.	n.d.	n.d.	0.77 ±0.01 ^a^	4.34 ±0.06 ^b^	0.4 8±0.01 ^a^	2.77 ±0.05 ^a^	1.74 ±0.03 ^c,d^	1.18 ±0.02 ^b^	11.54 ±0.13 ^f^	5.76 ±0.07 ^d^	n.d.	n.d.	n.d.	n.d.	4.06 ±0.06 ^g^	6.08 ±0.08 ^i^
	**256**	n.d.	n.d.	n.d.	n.d.	n.d.	n.d.	n.d.	1.00 ±0.01 ^a^	0.60 ±0.01 ^a^	1.58 ±0.02 ^a^	n.d.	0.013 ±0.006	n.d.	1.19 ±0.02 ^a^	n.d.	1.43 ±0.02 ^c^	1.21 ±0.02 ^d^
	**587**	n.d.	n.d.	n.d.	n.d.	n.d.	n.d.	n.d.	n.d.	n.d.	n.d.	n.d.	n.d.	n.d.	n.d.	n.d.	n.d.	n.d.
	**398**	n.d.	n.d.	n.d.	n.d.	n.d.	n.d.	n.d.	n.d.	n.d.	n.d.	n.d.	n.d.	n.d.	n.d.	n.d.	n.d.	n.d.
	**927**	n.d.	n.d.	n.d.	n.d.	n.d.	n.d.	n.d.	n.d.	n.d.	n.d.	n.d.	n.d.	n.d.	n.d.	n.d.	n.d.	n.d.
	**416**	n.d.	n.d.	n.d.	n.d.	n.d.	1.11 ±0.02 ^c^	4.29 ±0.06 ^b^	3.78 ±0.05 ^e^	11.81 ±0.12 ^h^	6.56 ±0.08 ^e^	13.22 ±0.14 ^g^	n.d.	n.d.	n.d.	n.d.	3.50 ±0.05 ^e^	0.95 ±0.01 ^c^
	**127**	n.d.	n.d.	0.82 ±0.01 ^a^	0.91 ±0.01 ^b^	n.d.	n.d.	n.d.	n.d.	n.d.	1.79 ±0.03 ^a^	1.58 ±0.02 ^a^	n.d.	n.d.	n.d.	n.d.	n.d.	n.d.
	**901**	n.d.	n.d.	n.d.	2.00 ±0.02 ^c^	1.57 ±0.02 ^a^	0.76 ±0.01 ^b^	2.75 ±0.03 ^a^	1.53 ±0.01 ^c^	9.31 ±0.09 ^g^	12.82 ±0.13 ^g^	3.05 ±0.03 ^c^	n.d.	n.d.	n.d.	n.d.	3.89 ±0.04 ^f^	3.60 ±0.03 ^f^
	**612**	n.d.	n.d.	1.13 ±0.01 ^b^	n.d.	n.d.	n.d.	n.d.	n.d.	3.40 ±0.03 ^e^	3.67 ±0.04 ^b^	1.64 ±0.01 ^a^	n.d.	n.d.	n.d.	n.d.	1.59 ±0.01 ^c^	1.13 ±0.01 ^d^
	**209**	2.68 ±0.04 ^b^	3.76 ±0.06	1.98 ±0.04 ^c^	1.95 ±0.03 ^c^	5.30 ±0.07	0.91 ±0.01 ^b^	6.32 ±0.08 ^d^	3.86 ±0.04 ^e^	1.95 ±0.03 ^c^	30.33 ±0.21 ^i^	26.07 ±0.15 ^h^	n.d.	n.d.	n.d.	n.d.	5.02 ±0.07 ^h^	5.03 ±0.07^h^
	**565**	2.07 ±0.02 ^a^	n.d.	3.09 ±0.03 ^d^	3.25 ±0.03 ^d^	n.d.	5.06 ±0.06 ^d^	4.61 ±0.06 ^c^	1.20 ±0.01 ^b^	25.27 ±0.18 ^i^	27.89 ±0.19 ^h^	7.93 ±0.09 ^e^	n.d.	n.d.	n.d.	n.d.	5.36 ±0.06 ^i^	4.78 ±0.04 ^g^
**BV**	**809**	n.d.	n.d.	n.d.	n.d.	n.d.	n.d.	n.d.	n.d.	n.d.	n.d.	n.d.	n.d.	n.d.	n.d.	n.d.	n.d.	n.d.
	**101**	n.d.	n.d.	n.d.	n.d.	n.d.	n.d.	n.d.	n.d.	n.d.	n.d.	n.d.	n.d.	n.d.	n.d.	n.d.	n.d.	n.d.
	**326**	n.d.	n.d.	n.d.	n.d.	n.d.	n.d.	n.d.	0.79 ±0.01 ^a^	0.40 ±0.01 ^a^	1.47 ±0.02 ^a^	n.d.	n.d.	n.d.	n.d.	n.d.	n.d.	n.d.
**PWV**	**834**	n.d.	n.d.	n.d.	n.d.	n.d.	n.d.	n.d.	n.d.	2.64 ±0.02 ^d^	4.49 ±0.05 ^c^	2.82 ±0.03 ^c^	n.d.	n.d.	n.d.	n.d.	0.91 ±0.01 ^a^	0.65 ±0.01 ^b^
	**215**	n.d.	n.d.	n.d.	n.d.	n.d.	n.d.	n.d.	n.d.	n.d.	n.d.	n.d.	n.d.	n.d.	n.d.	n.d.	n.d.	n.d.
	**462**	n.d.	n.d.	n.d.	n.d.	n.d.	n.d.	n.d.	n.d.	n.d.	n.d.	n.d.	n.d.	n.d.	n.d.	n.d.	1.17 ±0.01 ^b^	0.24 ±0.01 ^a^
	**198**	n.d.	n.d.	n.d.	n.d.	n.d.	n.d.	n.d.	n.d.	n.d.	n.d.	n.d.	n.d.	n.d.	n.d.	n.d.	n.d.	n.d.
	**703**	n.d.	n.d.	n.d.	n.d.	n.d.	n.d.	n.d.	n.d.	n.d.	n.d.	n.d.	n.d.	n.d.	n.d.	n.d.	n.d.	n.d.
	**619**	n.d.	n.d.	n.d.	n.d.	n.d.	n.d.	n.d.	n.d.	n.d.	n.d.	n.d.	n.d.	n.d.	n.d.	n.d.	n.d.	n.d.
***p***		<0.001	<0.001	<0.001	<0.001	<0.001	<0.001	<0.001	<0.001	<0.001	<0.001	<0.001	<0.001	n.d.	<0.001	n.d.	<0.001	<0.001

n.d.—not detected. * Aspartic Acid (Asp); Glutamic Acid (Glu); Asparagine (Asn); Serine (Ser); Histidine (Hist); Glutamine (Gln); Glycine (Gly); Threonine (Thr); Arginine (Arg); Alanine (Ala); Tyrosine (Tyr); Valine (Val); Methionine (Met); Tryptophan (Tryp); Phenylalanine (Phe); Isoleucine (Ile); Leucine (Leu).

**Table 3:** Daily intake of amino acids in 10 mL of wine vinegar versus recommended daily allowance (RDA), considering an adult of 50 kg. n.d.—not determined.

Amino Acids	Molar Mass (g/mol)	Daily Intake (mmol/L) per 10 mL of Sample 059	Classification	RDA for Adults mg/kg/Day [41]	Daily Intake for an Adult of 50 kg mg/50 kg/Day
**Aspartic Acid**	133.10	0.0766	Dispensable	n.d.	n.d.
**Glutamic acid**	147.13	0.0571	Dispensable	n.d.	n.d.
**Asparagine**	132.12	0.0386	Dispensable	n.d.	n.d.
**Serine**	105.09	0.0331	Dispensable	n.d.	n.d.
**Histidine**	155.16	0.1933	Indispensable	8–12	14.99
**Glutamine**	146.14	0.2106	Conditionally Indispensable	n.d.	n.d.
**Glycine**	75.07	0.1101	Conditionally Indispensable	n.d.	n.d.
**Threonine**	119.12	0.7147	Indispensable	7	42.56
**Arginine**	174.20	0.6121	Conditionally Indispensable	n.d.	n.d.
**Alanine**	89.09	0.1133	Dispensable	n.d.	n.d.
**Tyrosine**	181.19	0.1257	Conditionally Indispensable	n.d.	n.d.
**Valine**	117.15	0.0174	Indispensable	3.5	1.02
**Methionine**	149.21	0.0664	Indispensable	n.d.	n.d.
**Tryptophan**	204.23	0.3054	Indispensable	5	31.18
**Phenylalanine**	165.19	0.1911	Indispensable	n.d.	n.d.
**Isoleucine**	131.17	0.1361	Indispensable	10	8.92
**Leucine**	131.17	0.0964	Indispensable	14	6.32

## Data Availability

The original contributions presented in the study are included in the article, further inquiries can be directed to the corresponding author.
